# A Novel Modulator of STIM2-Dependent Store-Operated Ca2+ Channel Activity

**DOI:** 10.32607/actanaturae.11269

**Published:** 2021

**Authors:** A. Y. Skopin, A. D. Grigoryev, L. N. Glushankova, A. V. Shalygin, G. Wang, V. G. Kartzev, E. V. Kaznacheyeva

**Affiliations:** Institute of Cytology of Russian Academy of Sciences, St. Petersburg, 194064 Russia; College of Pharmaceutical Sciences, Soochow University, Suzhou, Jiangsu, 215123 China; InterBioscreen Ltd., Chernogolovka, 142432 Russia

**Keywords:** calcium, store-operated Ca2+ entry, STIM1, STIM2, 2-APB, Orai

## Abstract

Store-operated Ca^2+^ entry is one of the main pathways of calcium influx into non-excitable cells, which entails the initiation of many intracellular processes. The endoplasmic reticulum Ca^2+^ sensors STIM1 and STIM2 are the key components of store-operated Ca^2+^ entry in mammalian cells. Under physiological conditions, STIM proteins are responsible for store-operated Ca^2+^ entry activation. The STIM1 and STIM2 proteins differ in their potency for activating different store-operated channels. At the moment, there are no selective modulators of the STIM protein activity. We screened a library of small molecules and found the 4-MPTC compound, which selectively inhibited STIM2-dependent store-operated Ca^2+^ entry (*IC*50 = 1 μM) and had almost no effect on the STIM1-dependent activation of store-operated channels.

## INTRODUCTION

An increase in the concentration of cytoplasmic Ca^2+^ ions is one of the common cellular responses to extracellular stimulation of membrane receptors by physiologically active substances that trigger a wide range of intracellular cascades. Under physiological conditions, the intracellular Ca^2+^ response to an agonist includes not only entry of extracellular Ca^2+^ into the cell, but also depletion of the intracellular Ca^2+^ stores located in the endoplasmic reticulum (ER) [1]. Plasma membrane channel-mediated Ca^2+^ entry into the cell in response to the depletion of intracellular Ca^2+^ stores or store-operated Ca^2+^ entry [2] provides a significant part of the Ca^2+^ ion influx into the cell. The entry is induced by STIM proteins (STIM1 and STIM2), which are Ca^2+^ sensors in the ER lumen. The STIM1 protein, which is the main activator of store-operated Ca^2+^ entry, was the first to be characterized [3, 4]. The STIM1 and STIM2 proteins differ in their affinity for Ca^2+^ ions and ability to interact with plasma membrane channels [5]. STIM2 is more sensitive to small changes in the concentration of stored Ca^2+^ and is a weaker activator of store-operated Ca^2+^ entry than STIM1. STIM1 is most likely responsible for the cellular Ca^2+^ response to an extracellular signal, while STIM2 regulates the basal levels of cytosolic and stored Ca^2+^ [6]. In addition, STIM2 facilitates STIM1 transition to the active state [7]. Under physiological conditions, STIM1 and STIM2 activate various store-operated channels in the cell [8], which are formed by proteins belonging to the Orai [9, 10] and TRP [11, 12, 13] families. STIM proteins are involved in a wide range of pathologies. For instance, a long-term increase in the neuronal Ca^2+^ concentration, which is caused by an enhanced activity of STIM proteins and leads to cell death, is observed in Huntington’s disease [14, 15], Alzheimer’s disease [16, 17], cerebral ischemia [18], and traumatic brain injury [19, 20]. Changes in STIM expression levels are typical for several breast cancers [21] and colon carcinoma [22]. Thus, changes in the activity of STIM proteins, in particular decreased STIM2 activity, may possess a potential therapeutic effect. In basic research, a STIM2 activity modulator would be an essential tool to be used to distinguish between STIM1- and STIM2-mediated signaling pathways, because such pharmacological agents are currently unavailable.

Researchers have actively used a wide range of store-operated Ca^2+^ entry inhibitors. Most of these inhibitors modulate the activity of store-operated Ca^2+^ channels. However, these compounds are often poorly characterized and have more than one target. One of the most commonly used compounds, 2-aminoethoxydiphenyl borate (2-APB), was first characterized as a blocker of IP3-induced Ca^2+^ release [23]. It is now widely used as a store-operated Ca^2+^ entry inhibitor at concentrations exceeding 50 μM. In addition, 2-APB, at a concentration of 5 μM, can potentiate store-operated entry [24]. The mechanism of 2-APB action is not fully understood; this compound is known to have several targets and, in particular, to exert a modulatory effect on the activity of various channels; e.g., TRPV [25, 26] and Orai3 [27] channels. The 2-APB compound also enhances non-specific Ca^2+^ leak from the ER lumen [28].

When ER Ca^2+^ stores are filled, STIM proteins are in an inactive conformation stabilized by the interaction between the CC1 (Coiled-Coil 1) and SOAR (STIMOrai Activating Region) domains. Following Ca^2+^ store depletion, STIM proteins undergo multimerization, change their conformation, and expose the SOAR domain for interaction with plasma membrane channels [29]. The 2-APB compound, at concentrations of about 10 μM, is known to induce store-operated Ca^2+^ entry by transforming STIM2 into its active conformation [30]. On the contrary, 2-APB at a higher concentration (50 μM) stabilizes an inactive STIM1 conformation by enhancing the interaction between the CC1 and SOAR domains. Thus, it inhibits the interaction of the SOAR domain with Orai1 channels and the activation of the channels. Interestingly, increased Orai1 expression partially reverses this action [31].

Thus, 2-APB directly interacts with STIM proteins and provides a good basis for the search for a more selective modulator of store-operated Ca^2+^ entry. In this work, we have tested a library of 250 chemical compounds received from InterBioScreen Ltd. possessing a chemical structure similar to that of 2-APB, in order to identify a selective modulator of STIM2 activity. A 4-MPTC compound was found to inhibit STIM2- dependent Ca^2+^ entry (*IC*50 = 1 μM) but had almost no effect on the STIM1-mediated mechanism of storeoperated channel activation. The other 249 compounds from the library had a divergent, and non-selective, effect.

## EXPERIMENTAL

**Cell lines**

The following HEK293-derived cell lines, kindly provided by Jonathan Soboloff and Mohamed Trebak, were used in the study: STIM1Orai3 (a cell line expressing exogenous STIM1-YFP and Orai3-CFP proteins), STIM2Orai3 (a cell line expressing exogenous STIM2-YFP and Orai3-CFP proteins) [32], STIM1 KO (a CRISPR/Cas9-mediated STIM1 gene knockout cell line), STIM2 KO (a CRISPR/Cas9-mediated STIM2 gene knockout cell line), and Orai3 KO (a CRISPR/Cas9-mediated Orai3 knockout cell line) [30]. The cell lines were cultured in a DMEM medium (Biolot, Russia) supplemented with 10% fetal bovine serum, as well as the antibiotics penicillin (100 U/ml) and streptomycin (0.1 mg/ml) at 37°C and 5% CO_2_.

**Fluorescence analysis**

Changes in the intracellular Ca^2+^ concentration were measured using a Fluo-4 AM calcium indicator (Thermo Fisher Scientific, USA). The cells were plated into 96-well culture plates 48 h prior to the analysis. The cells were first incubated in a HBSS solution (2 mM CaCl_2_, 130 mM NaCl, 25 mM KCl, 1.2 mM MgCl_2_, 10 mM HEPES, and 10 mM glucose) containing 5 μM Fluo-4 AM for 1 h and then in a HBSS solution supplemented with either 4-MPTC (InterBioScreen Ltd., Russia) or 1% DMSO (Sigma-Aldrich, USA) for 30 min. Measurements were performed in the presence of 2 mM calcium in the extracellular solution using a FLUOstar Omega microplate reader (BMG Labtech, Germany). Data are presented as Fluo-4 fluorescence intensity values normalized to the basal fluorescence value.

**Electrophoresis and immunoblotting**

The cells were grown in 60-mm Petri dishes and then lysed by adding a protease inhibitor cocktail. Proteins were separated by 8% denaturing PAGE. The proteins were transferred to a nitrocellulose membrane using a semi-dry transfer unit (Hoefer Pharmacia Biotech., Germany). Primary antibodies to STIM1 (Cell Signaling #4917, USA), STIM2 (Cell Signaling #5668, USA), and α-tubulin (Sigma-Aldrich #T6074, USA) were diluted at a ratio of 1 : 1000. Next, secondary anti-mouse IgG antibodies (Sigma-Aldrich #A0168, USA) against α-tubulin and anti-rabbit IgG antibodies (Sigma- Aldrich #A0545, USA) against STIM1 and STIM2 were used. Blots were visualized on a BioRad Cell Imaging System (Bio-Rad Laboratories, Inc., USA).

Low-molecular-weight compounds for screening, including 4-MPTC, were kindly provided by InterBio- Screen Ltd. (ibscreen.com) in dry form. The compounds were dissolved in DMSO to a final concentration of 10 mM.

**Statistical analysis**

Statistical analysis was performed using the Origin 8 software. The results of fluorescence measurements were checked for normality using the Fisher’s test. Data groups were compared using the Bonferroni test. Statistically significant differences are denoted in figures as follows: “*”–the confidence interval of *p* < 0.05, “**”–*p* < 0.01, “***”–*p* < 0.001; “n.s.” –not statistically significant differences.

## RESULTS AND DISCUSSION

**Fig. 1:**
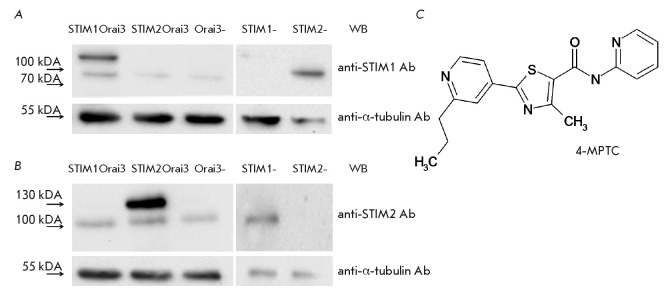
Expression levels of STIM proteins in the STIM1Orai3, STIM2Orai3, Orai3 KO, STIM1 KO, and STIM2 KO cell lines. (*A*) Western blot using anti-STIM1 antibodies. (*B*) Western blot using anti-STIM2 antibodies. Anti-α-tubulin antibodies were used as a control to assess the uniformity of sample loading. (*C*) Structural formula of 4-MPTC

**Fig. 2:**
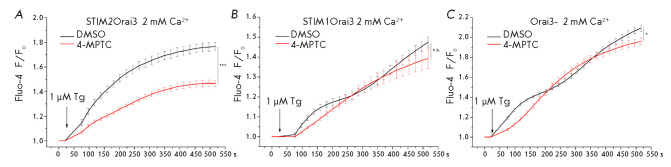
Effect of the 4-MPTC compound on the Tg-induced calcium response. Measurements were performed in cell lines with (*A*) exogenous STIM2 and Orai3 protein expression, (*B*) exogenous STIM1 and Orai3 protein expression, and (*C*) Orai3 protein knockout. The dependence of Fluo-4 fluorescence, normalized to the basal fluorescence level, on time is presented. Prior to starting the experiment, the cells were incubated in HBSS supplemented with 100 μM 4-MPTC for 30 min. Control cells were incubated in HBSS containing 1% DMSO for 30 min. Data are presented as means ± s.e.m. (n = 12)

In order to search for low-molecular-weight compounds that modulate the activity of STIM2 proteins, we used a model cell line derived from HEK293 cells stably expressing exogenous STIM2 and Orai3 proteins (STIM2Orai3 cell line) (*Fig. 1A*). The effect of the test compounds on the amplitude of a cellular Ca^2+^ signal in response to the depletion of intracellular Ca^2+^ stores was recorded using the Fluo-4 AM calcium indicator. Intracellular Ca^2+^ stores were depleted by adding 1 μM thapsigargin (Tg), a selective inhibitor of the ER Ca^2+^ pump, to the extracellular solution. At the first stage, the effect of the library of 2-APB analogs on the Tg-induced Ca^2+^ response was tested. For this purpose, the cells were incubated in HBSS solutions containing one of the 250 test compounds (at a concentration of 100 μM) for 30 min prior to starting the experiments. Next, the amplitude of the Ca^2+^ response to the addition of 1 μM Tg was assessed. As a result of library screening, we selected 4-MPTC (*Fig. 1C*), the compound that most strongly affected the Tg-induced Ca^2+^ response in STIM2Orai3 cells: the Ca^2+^ response was inhibited by 39 Ѓ} 3% compared to that in the cells incubated in a solution supplemented with 1% DMSO (*Fig. 2A*). Since 4-MPTC significantly inhibits the Tg-induced Ca^2+^ response in cells with increased STIM2 and Orai3 levels, we may suggest that 4-MPTC modulates the activity of these proteins. The direct action of 4-MPTC on Orai3 is supported by the fact that 2-APB can activate the Orai3 channel [27]. To test the effect of 4-MPTC on Orai3 channels, HEK293 cells with Orai3 knockout (the Orai3 KO cell line) were used. Incubation of Orai3 KO cells with 4-MPTC changed the shape of the Tg-induced Ca^2+^ response and decreased its amplitude by 12 Ѓ} 3% (*Fig. 2B*). Furthermore, incubation of HEK293 cells expressing exogenous STIM1 and Orai3 proteins (the STIM1Orai3 cell line) with 4-MPTC did not inhibit the amplitude of the Tg-induced Ca^2+^ response (*Fig. 2B*) and, therefore, did not decrease the activity of the Orai3 channels. Hence, the Orai3 protein is not a selective target for 4-MPTC.

The activity of store-operated channels in a cell is known to be modulated by both the STIM1 and STIM2 proteins [8]. The predominant pathway of store-operated entry activation can be modulated through either the STIM1 protein or the STIM2 protein by changing their expression levels. HEK293 cells expressing exogenous STIM1 and Orai3 proteins were used to test the effect of 4-MPTC on STIM1. As mentioned above, incubation of STIM1Orai3 cells with 4-MPTC changes the shape of the Tg-induced Ca^2+^ response without decreasing its amplitude (*Fig. 2B*). Since 4-MPTC significantly reduced the Ca^2+^ response amplitude but did not alter the curve’s shape in STIM2Orai3 cells (*Fig. 2A*), we may suggest that this compound affects the pathway of store-operated calcium entry activation through STIM2, but not through the STIM1 protein. A change in the curve’s shape for the Orai3 KO and STIM1Orai3 cell lines is quite typical and reflects a decrease in the rate of the Ca^2+^ response. Since the endogenous STIM2 protein is present in Orai3 KO and STIM1Orai3 cells (*Fig. 1B*), 4-MPTC can reduce its activity and, thereby, change the dynamics of both the release of Ca^2+^ from the store into the cytoplasm and the entry of extracellular Ca^2+^ ions. Knockout of STIM2 using short interfering RNAs results in a similar effect on the Ca^2+^ response; it decreases Ca^2+^ release from the store [33] and subsequent Ca^2+^ entry [4, 34]. Cell lines overexpressing STIM proteins (STIM1Orai3 and STIM2Orai3) contain endogenous STIM1 and STIM2 (*Fig. 1A,B*), which complicates data interpretation. Therefore, we further used STIM1 (the STIM1 KO cell line) and STIM2 knockout cells (the STIM2 KO cell line), which are devoid of this drawback (*Fig. 1A,B*).

**Fig. 3:**
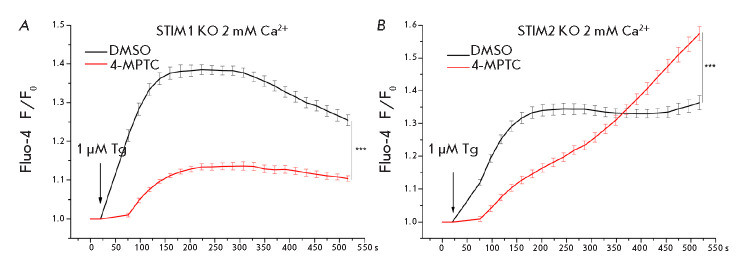
Effect of the 4-MPTC compound on the Tg-induced calcium response. Measurements were performed in (*A*) STIM1 knockout and (*B*) STIM2 knockout cells. The dependence of the Fluo-4 fluorescence, normalized to the basal fluorescence level, on time is presented. Prior to starting the experiment, the cells were incubated in HBSS supplemented with 100 μM 4-MPTC for 30 min. Control cells were incubated in HBSS containing 1% DMSO for 30 min. Data are presented as means ± s.e.m. (n = 12)

When STIM1 expression is completely suppressed, the STIM2 protein becomes the key and only activator of store-operated Ca^2+^ entry [4]. Pre-incubation of STIM1 KO cells with 4-MPTC decreased the Tginduced Ca^2+^ response by 57 ± 8% compared to that in control cells (incubation with 1% DMSO) (*Fig. 3A*). It should be noted that 4-MPTC more effectively inhibits store-operated Ca^2+^ entry under these conditions. For example, the Tg-induced Ca^2+^ response was inhibited by 57% in STIM1 KO cells and by only 39% in STIM2Orai3 cells. A significant change in the shape of the Tg-induced response is observed after incubation of STIM2-knockout cells in which the STIM1 protein is the only activator of store-operated Ca^2+^ entry with 4-MPTC. The calcium concentration increases more slowly in these cells than in the control cells, with the maximum Ca^2+^ response amplitude being 61 Ѓ} 5% higher compared to that in the control (*Fig. 3B*). 4-MPTC was experimentally demonstrated to act divergently in STIM1 KO and STIM2 KO cell lines: it inhibits the Ca^2+^ response through the STIM2-dependent pathways and enhances it through the STIM1 pathways. Thus, the selected compound, 4-MPTC, enables differentiation between the pathways activating store-operated Ca^2+^ entry through different STIM proteins; however, the mechanism of action of this compound requires further clarification.

**Fig. 4:**
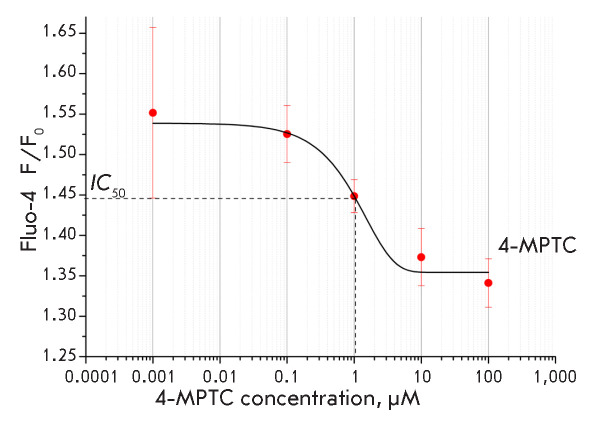
Dependence of the Tg-induced calcium response amplitude on the 4-MPTC concentration. Measurements were performed in a cell line expressing exogenous STIM2 and Orai3 proteins. The Fluo-4 fluorescence intensity at minute 9 of the experiment, normalized to the basal fluorescence level, is presented. Prior to the experiment, the cells were incubated at various concentrations of 4-MPTC (0.001, 0.1, 1, 10, and 100 μM) for 30 min. Data are presented as means ± s.e.m. (n = 6). The half-maximum inhibitory level and half-maximum inhibitory concentration (*IC*50 = 1 rKM) are denoted by dotted lines

The 4-MPTC compound has a typical concentration– effect curve (*Fig. 4*). We analyzed the effect of 4-MPTC at a concentration of 0.001, 0.1, 1, 10, and 100 μM on the Tg-induced Ca^2+^ response in STIM2Orai3 cells. The half-maximal inhibitory concentration (*IC*50), calculated by curve-fitting, equals 1 μM.

Thus, given our findings, we may conclude that the use of 4-MPTC in cell lines expressing predominantly the STIM2 protein (STIM1 KO and STIM2Orai3) significantly inhibits the amplitude of the Tg-induced Ca^2+^ response, while the use of 4-MPTC in cell lines producing predominantly the STIM1 protein (STIM2 KO, STIM1Orai3), on the contrary, changes the shape of the Ca^2+^ response curve, without decreasing its amplitude. Thus, 4-MPTC selectively inhibits storeoperated Ca^2+^ entry via the STIM2-mediated pathway, but not the STIM1-mediated pathway.

Despite the fact that 2-APB is widely used as a store-operated Ca^2+^ entry inhibitor, it does not appear to selectively inhibit store-operated Ca^2+^ entry and also has a divergent concentration-dependent effect. 2-APB derivatives have been investigated in the search for an inhibitor lacking these disadvantages [35-41]. Most of the identified compounds inhibit store-operated Ca^2+^ entry at lower concentrations than 2-APB but are at the same time unable to activate Ca^2+^ entry at certain concentrations; in other words, they have better inhibitory properties than the parent compound. More attention in the search for new inhibitors of store-operated Ca^2+^ entry has been paid to the STIM1-dependent pathway of activation, while the STIM2-mediated pathway often has remained unexplored. For example, MDA-MB-231 cells, in which STIM1 and Orai1 proteins play a key role in store-operated Ca^2+^ entry, as well as HEK293 cells expressing STIM1 and Orai-family proteins, have been used as model cell lines in experiments [42, 43]. A study of the compounds DPB163-AE and DPB162-AE demonstrated that they interact differently with STIM1 and STIM2 but eventually inhibit store-operated Ca^2+^ entry through both proteins [37]. The 4-MPTC compound, identified in our study, has an inhibitory effect on the STIM2-mediated pathway and does not inhibit Ca^2+^ entry through the STIM1- dependent pathway.

## CONCLUSION

Screening of a library of structure 2-APB analogs has yielded an 4-MPTC compound that has an inhibitory effect on the Tg-induced Ca^2+^ response through the STIM2-dependent pathway of Ca^2+^ influx but does not inhibit Ca^2+^ entry through the STIM1-dependent pathway. The mechanism of action of this compound on the STIM2 protein requires further investigation.

## Abbreviations

2-APB: 2-aminoethoxydiphenyl borate
4-MPTC: 4-methyl-2-(2-propylpyridin-4-yl)-N- (pyridin-2-yl)thiazole-5-carboxamide
CC1: coiled-coil 1 domain
DMSO: dimethyl sulfoxide
STIM: stromal- interacting molecule
SOAR: STIM-ORAI activating region
Tg: thapsigargin
ER: endoplasmic reticulum.
